# [μ-*N*,*N*,*N*′,*N*′-Tetra­kis­(2-pyridyl­meth­yl)pentane-1,5-diamine]­bis­[dichlorido­copper(II)] sesquihydrate

**DOI:** 10.1107/S1600536810034513

**Published:** 2010-09-04

**Authors:** Mark Bartholomä, Hoi Cheung, Kari Darling, Jon Zubieta

**Affiliations:** a Department of Chemistry, Syracuse University, Syracuse, New York 13244, USA

## Abstract

In the title dinuclear copper complex, [Cu_2_Cl_4_(C_29_H_34_N_6_)]·1.5H_2_O, both Cu^II^ ions are coordinated in a slightly distorted square-pyramidal environment in which the N atoms of the dipicolyl­amine group and a chloride ligand form the basal plane. The apical position is occupied by a second chloride atom. The Cu—N distances involving the pyridine N atoms differ slightly from each other and the Cu—N distance involving the tertiary N atom is the longest. The apical Cu—Cl distance is elongated compared to its basal counterpart due to typical Jahn–Teller distortion. In the crystal structure, complex and water mol­ecules are linked *via* inter­molecular O—H⋯O and O—H⋯Cl hydrogen bonds into chains along [001]. One of the water mol­ecules was refined with half occupancy.

## Related literature

For crystallographic data of tetra­kis­(pyridin-2-yl-meth­yl)alkyl-diamines, see: Fujihara *et al.* (2004 ▶); Mambanda *et al.* (2007 ▶). For the superoxide dismutase activity of iron complexes, see: Tamura *et al.* (2000 ▶). For dinuclear Pt complexes of similar ligands, see: Ertürk *et al.* (2007 ▶). For the use of the dipicolyl­amine moiety for binding of the *M*(CO)_3_ core (*M* = Re, ^99*m*^Tc), see: Bartholomä *et al.* (2009 ▶). For crystal structures closely related to the title compound, see: Bartholomä *et al.* (2010*a* ▶,*b* ▶,*c* ▶,*d* ▶).
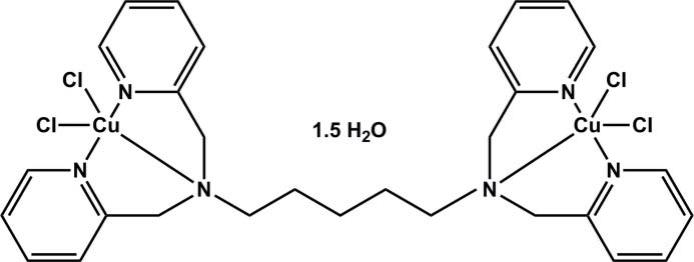


## Experimental

### 

#### Crystal data

[Cu_2_Cl_4_(C_29_H_34_N_6_)]·1.5H_2_O
*M* *_r_* = 762.55
Monoclinic, 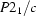

*a* = 8.2003 (13) Å
*b* = 14.3700 (16) Å
*c* = 27.688 (4) Å
β = 97.919 (6)°
*V* = 3231.6 (8) Å^3^
*Z* = 4
Mo *K*α radiation
μ = 1.68 mm^−1^
*T* = 90 K
0.22 × 0.16 × 0.12 mm

#### Data collection

Bruker APEX CCD diffractometer
Absorption correction: multi-scan (*SADABS*; Sheldrick, 1996 ▶) *T*_min_ = 0.709, *T*_max_ = 0.824
31227 measured reflections
7827 independent reflections
7421 reflections with *I* > 2σ(*I*)
*R*_int_ = 0.050

#### Refinement

*R*[*F* ^2^ > 2σ(*F* ^2^)] = 0.079
*wR*(*F* ^2^) = 0.156
*S* = 1.40
7827 reflections
404 parameters
5 restraints
H atoms treated by a mixture of independent and constrained refinement
Δρ_max_ = 0.81 e Å^−3^
Δρ_min_ = −0.67 e Å^−3^

### 

Data collection: *SMART* (Bruker, 2002 ▶); cell refinement: *SAINT* (Bruker, 2002 ▶); data reduction: *SAINT*; program(s) used to solve structure: *SHELXS97* (Sheldrick, 2008 ▶); program(s) used to refine structure: *SHELXL97* (Sheldrick, 2008 ▶); molecular graphics: *DIAMOND* (Brandenburg & Putz, 1999 ▶); software used to prepare material for publication: *SHELXTL* (Sheldrick, 2008 ▶).

## Supplementary Material

Crystal structure: contains datablocks I, global. DOI: 10.1107/S1600536810034513/lh5109sup1.cif

Structure factors: contains datablocks I. DOI: 10.1107/S1600536810034513/lh5109Isup2.hkl

Additional supplementary materials:  crystallographic information; 3D view; checkCIF report

## Figures and Tables

**Table 1:** Selected bond lengths (Å)

Cu1—N1	1.986 (4)
Cu1—N3	1.996 (4)
Cu1—N2	2.076 (4)
Cu1—Cl2	2.2832 (13)
Cu1—Cl1	2.5261 (13)

**Table 2:** Hydrogen-bond geometry (Å, °)

*D*—H⋯*A*	*D*—H	H⋯*A*	*D*⋯*A*	*D*—H⋯*A*
O2—H2*WB*⋯Cl1^i^	0.83 (2)	2.54 (7)	3.270 (8)	147 (11)
O2—H2*WA*⋯O1^ii^	0.84 (2)	2.46 (4)	3.249 (11)	157 (10)
O1—H1*WB*⋯Cl2^iii^	0.84 (2)	2.54 (4)	3.335 (6)	159 (8)
O1—H1*WA*⋯Cl4^iv^	0.84 (2)	2.47 (3)	3.306 (6)	169 (10)

Symmetry codes: (i) 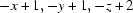; (ii) 
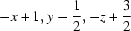
; (iii) 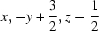; (iv) 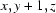.

**Fig. 1.**
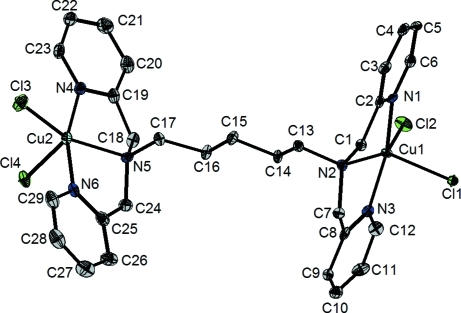
The molecular structure of the title complex. The displacement ellipsoids are drawn at 50% probability level. solvent water molecules and hydrogen atoms are omitted for clarity.

## supplementary crystallographic information

### Comment

The described ligand has been used as starting material for hydrothermal synthesis of metal-organic transition metal/molybdateoxide frameworks in the principal author's laboratory (Bartholomä, unpublished results). The dipicolylamine moiety has originally been developed in our laboratory as metal chelating entity for binding of the *M*(CO)_3_ core (*M* = Re,^99^*^m^*Tc) for radiopharmaceutical purposes. However, a different coordination mode has been observed for the *M*(CO)_3_ core in which the dipicolylamine metal chelate is coordinated in a facial manner (Bartholomä, 2009).

The title complex was prepared as part of a series with different cadmium and copper salts to study the coordination properties of the ligand with these metals without the interaction of metaloxide clusters (Bartholomä, 2010*a*,b,d). The shorter homologue *N^1^*,*N^1^*,*N^4^*,*N^4^*-tetrakis(pyridin-2-ylmethyl)butane-1,4-diamine gave structurally similar complexes with copper bromide as metal salt. The corresponding Cu—N_py_ distances are 2.015 (6) Å and 2.019 (6) Å and the Cu—N*_tert_* distance was determined to 2.053 (5) Å (Bartholomä, 2010*c*).

Crystal structures of the ligands *N^1^*,*N^1^*,*N^3^*,*N^3^*-tetrakis(2-pyridiniomethyl)-1,3-diaminopropane and *N^1^*,*N^1^*,*N^4^*,*N^4^*-tetrakis(pyridin-2-ylmethyl)butane-1,4-diamine have been described recently (Fujihara, 2004; Mambanda, 2007). Superoxide dismutase activity of iron(II) complexes of *N^1^*,*N^1^*,*N^3^*,*N^3^*-tetrakis(2-pyridiniomethyl)-1,3-diaminopropane and related ligands has been investigated by Tamura *et al.* (2000). Studies on the thermodynamic and kinetic behaviour of the reaction of platinum(II) complexes of higher ligand homologues with chloride have been performed by Ertürk *et al.* (2007).

### Experimental

*N^1^*,*N^1^*,*N^5^*,*N^5^*-tetrakis(pyridin-2-ylmethyl)pentane-1,5-diamine. An amount of 1.00 g (9.78 mmol) 1,5-diaminopentane was dissolved in 30 ml anhydrous dichloroethane under an inert atmosphere (argon) followed by the addition of 3.91 ml (41.10 mmol) pyridine-2-carboxaldehyde. The mixture was stirred for 15 min at r.t. and then cooled with an ice bath prior to the portionwise addition of 12.44 g (58.72 mmol) sodium triacetoxyborohydride (gas evolution, exothermic reaction). The reaction was stirred overnight allowing the temperature slowly to rise to room temperature. The reaction was quenched by the dropwise addition of saturated sodium bicarbonate solution and stirring was continued until the gas evolution ceased. The mixture was separated and the organic layer was further washed with saturated sodium bicarbonate solution, water and brine. The organic phase was dried with anhydrous sodium sulfate, filtered and the solvent removed under reduced pressure. The crude reaction mixture was then purified by silica gel column chromatography starting with chloroform and increasing gradient to chloroform:methanol 10:1 (*v*/*v*). Yield: 4.02 g (86%). ^1^H NMR (CDCl_3_): *δ* = 8.39 (m, 4H), 7.49 (m, 4H), 7.37 (d, J = 7.81 Hz, 4H), 6.95 (m, 4H), 3.64 (s, 8H), 2.36 (m, 4H), 1.33 (m, 4H), 1.07 (m, 2H) p.p.m..

Synthesis of metal complex. To 2 ml of an aqueous solution of copper chloride, two equivalents (50 mg, 0.11 mmol) of *N^1^*,*N^1^*,*N^5^*,*N^5^*-tetrakis(pyridin-2-ylmethyl)pentane-1,5-diamine in 2 ml methanol were added followed by the addition of 2 ml *N*,*N*-dimethylformamide. Single crystals were obtained after a week by slow evaporation of the solvents at room temperature.

### Refinement

All the H atoms were placed in idealized positions and refined by the riding model approximation with *C*—H_aryl_ = 0.95 Å, *C*—H_methyl_ = 0.98Å and *C*—H_methylene_ = 0.99Åand *U*_iso_(H) = 1.5*U*_eq_(C_methyl_) and 1.2*U*_eq_(C_methylene/aryl_). Water hydrogen atoms were initially located in the difference Fourier map but were then refined with O—H = 0.84 (2) Å and *U*_iso_(H) = 1.5 *U*_eq_(O).

### Crystal data

**Table:**

[Cu_2_Cl_4_(C_29_H_34_N_6_)]·1.5H_2_O	*F*(000) = 1564
*M**_r_* = 762.55	*D*_x_ = 1.561 Mg m^−^^3^
Monoclinic, *P*2_1_/*c*	Mo *K*α radiation, λ = 0.71073 Å
Hall symbol: -P 2ybc	Cell parameters from 6480 reflections
*a* = 8.2003 (13) Å	θ = 5.3–56.1°
*b* = 14.3700 (16) Å	µ = 1.68 mm^−^^1^
*c* = 27.688 (4) Å	*T* = 90 K
β = 97.919 (6)°	Neeldes, blue
*V* = 3231.6 (8) Å^3^	0.22 × 0.16 × 0.12 mm
*Z* = 4	

### Data collection

**Table:**

Bruker APEX CCD diffractometer	7827 independent reflections
Radiation source: fine-focus sealed tube	7421 reflections with *I* > 2σ(*I*)
graphite	*R*_int_ = 0.050
Detector resolution: 512 pixels mm^-1^	θ_max_ = 28.1°, θ_min_ = 1.6°
φ and ω scans	*h* = −10→10
Absorption correction: multi-scan (*SADABS*; Sheldrick, 1996)	*k* = −18→18
*T*_min_ = 0.709, *T*_max_ = 0.824	*l* = −36→35
31227 measured reflections	

### Refinement

**Table:**

Refinement on *F*^2^	Primary atom site location: structure-invariant direct methods
Least-squares matrix: full	Secondary atom site location: difference Fourier map
*R*[*F*^2^ > 2σ(*F*^2^)] = 0.079	Hydrogen site location: inferred from neighbouring sites
*wR*(*F*^2^) = 0.156	H atoms treated by a mixture of independent and constrained refinement
*S* = 1.40	*w* = 1/[σ^2^(*F*_o_^2^) + (0.0289*P*)^2^ + 16.2006*P*] where *P* = (*F*_o_^2^ + 2*F*_c_^2^)/3
7827 reflections	(Δ/σ)_max_ = 0.003
404 parameters	Δρ_max_ = 0.81 e Å^−^^3^
5 restraints	Δρ_min_ = −0.67 e Å^−^^3^

### Special details

**Table:**

Geometry. All e.s.d.'s (except the e.s.d. in the dihedral angle between two l.s. planes) are estimated using the full covariance matrix. The cell e.s.d.'s are taken into account individually in the estimation of e.s.d.'s in distances, angles and torsion angles; correlations between e.s.d.'s in cell parameters are only used when they are defined by crystal symmetry. An approximate (isotropic) treatment of cell e.s.d.'s is used for estimating e.s.d.'s involving l.s. planes.
Refinement. Refinement of *F*^2^ against ALL reflections. The weighted *R*-factor *wR* and goodness of fit *S* are based on *F*^2^, conventional *R*-factors *R* are based on *F*, with *F* set to zero for negative *F*^2^. The threshold expression of *F*^2^ > σ(*F*^2^) is used only for calculating *R*-factors(gt) *etc*. and is not relevant to the choice of reflections for refinement. *R*-factors based on *F*^2^ are statistically about twice as large as those based on *F*, and *R*- factors based on ALL data will be even larger.

### Fractional atomic coordinates and isotropic or equivalent isotropic displacement parameters (Å^2^)

**Table:**

	*x*	*y*	*z*	*U*_iso_*/*U*_eq_	Occ. (<1)
Cu1	0.27113 (7)	0.72996 (4)	1.01183 (2)	0.01076 (13)	
Cu2	0.27749 (7)	0.33032 (4)	0.71531 (2)	0.01413 (14)	
Cl1	0.18034 (14)	0.87113 (8)	1.05552 (4)	0.0141 (2)	
Cl2	0.37112 (16)	0.61583 (9)	1.06521 (5)	0.0213 (3)	
Cl3	0.41385 (17)	0.36546 (10)	0.65260 (5)	0.0258 (3)	
Cl4	0.10401 (16)	0.18600 (9)	0.69447 (5)	0.0215 (3)	
O1	0.2269 (9)	0.9799 (4)	0.6606 (2)	0.0570 (16)	
O2	0.9605 (10)	0.2886 (6)	0.8770 (3)	0.0253 (17)	0.50
N1	0.4863 (5)	0.7920 (3)	1.00962 (14)	0.0113 (8)	
N2	0.2270 (5)	0.7828 (3)	0.94143 (14)	0.0114 (8)	
N3	0.0433 (5)	0.6801 (3)	0.99475 (15)	0.0120 (8)	
N4	0.4603 (5)	0.2609 (3)	0.75526 (15)	0.0159 (8)	
N5	0.2081 (5)	0.3520 (3)	0.78308 (15)	0.0132 (8)	
N6	0.0870 (5)	0.4163 (3)	0.69621 (15)	0.0168 (9)	
C1	0.3151 (6)	0.8723 (3)	0.94483 (17)	0.0114 (9)	
H1A	0.2495	0.9199	0.9594	0.014*	
H1B	0.3315	0.8937	0.9118	0.014*	
C2	0.4794 (6)	0.8601 (3)	0.97598 (17)	0.0140 (9)	
C3	0.6144 (6)	0.9175 (3)	0.97175 (19)	0.0158 (10)	
H3	0.6075	0.9657	0.9480	0.019*	
C4	0.7587 (6)	0.9022 (4)	1.0032 (2)	0.0190 (11)	
H4	0.8526	0.9401	1.0012	0.023*	
C5	0.7659 (6)	0.8316 (4)	1.03749 (19)	0.0180 (10)	
H5	0.8642	0.8201	1.0591	0.022*	
C6	0.6271 (6)	0.7785 (3)	1.03951 (18)	0.0156 (9)	
H6	0.6314	0.7302	1.0631	0.019*	
C7	0.0454 (6)	0.7921 (3)	0.92979 (17)	0.0131 (9)	
H7A	0.0132	0.7887	0.8940	0.016*	
H7B	0.0108	0.8535	0.9411	0.016*	
C8	−0.0398 (6)	0.7164 (3)	0.95395 (17)	0.0132 (9)	
C9	−0.1994 (6)	0.6872 (3)	0.93578 (17)	0.0141 (9)	
H9	−0.2557	0.7132	0.9066	0.017*	
C10	−0.2733 (6)	0.6204 (3)	0.96078 (19)	0.0169 (10)	
H10	−0.3812	0.5992	0.9490	0.020*	
C11	−0.1882 (6)	0.5841 (4)	1.0036 (2)	0.0199 (11)	
H11	−0.2382	0.5388	1.0218	0.024*	
C12	−0.0301 (6)	0.6148 (3)	1.01933 (19)	0.0175 (10)	
H12	0.0286	0.5892	1.0483	0.021*	
C13	0.2943 (6)	0.7235 (3)	0.90419 (17)	0.0128 (9)	
H13A	0.4160	0.7265	0.9103	0.015*	
H13B	0.2590	0.7506	0.8716	0.015*	
C14	0.2430 (6)	0.6220 (3)	0.90307 (16)	0.0129 (9)	
H14A	0.1215	0.6173	0.8992	0.016*	
H14B	0.2880	0.5915	0.9341	0.016*	
C15	0.3081 (6)	0.5735 (4)	0.86064 (19)	0.0172 (10)	
H15A	0.4298	0.5711	0.8673	0.021*	
H15B	0.2780	0.6109	0.8307	0.021*	
C16	0.2422 (6)	0.4749 (3)	0.85142 (18)	0.0165 (10)	
H16A	0.1211	0.4746	0.8504	0.020*	
H16B	0.2902	0.4334	0.8782	0.020*	
C17	0.2879 (6)	0.4396 (3)	0.80292 (17)	0.0147 (9)	
H17A	0.2611	0.4891	0.7783	0.018*	
H17B	0.4086	0.4303	0.8069	0.018*	
C18	0.2662 (6)	0.2696 (3)	0.81311 (18)	0.0161 (10)	
H18A	0.1872	0.2176	0.8061	0.019*	
H18B	0.2738	0.2851	0.8482	0.019*	
C19	0.4314 (6)	0.2415 (3)	0.80118 (18)	0.0155 (9)	
C20	0.5471 (7)	0.1945 (4)	0.8336 (2)	0.0222 (11)	
H20	0.5277	0.1838	0.8662	0.027*	
C21	0.6906 (7)	0.1636 (4)	0.8181 (2)	0.0268 (12)	
H21	0.7702	0.1306	0.8397	0.032*	
C22	0.7170 (7)	0.1812 (4)	0.7709 (2)	0.0218 (11)	
H22	0.8136	0.1590	0.7593	0.026*	
C23	0.6002 (7)	0.2317 (4)	0.74057 (19)	0.0207 (11)	
H23	0.6204	0.2460	0.7084	0.025*	
C24	0.0260 (6)	0.3601 (4)	0.77349 (19)	0.0181 (10)	
H24A	−0.0155	0.3899	0.8017	0.022*	
H24B	−0.0239	0.2974	0.7689	0.022*	
C25	−0.0214 (6)	0.4176 (4)	0.72845 (19)	0.0192 (10)	
C26	−0.1696 (7)	0.4657 (4)	0.7199 (2)	0.0241 (12)	
H26	−0.2452	0.4649	0.7430	0.029*	
C27	−0.2036 (7)	0.5149 (4)	0.6766 (2)	0.0301 (14)	
H27	−0.3053	0.5470	0.6692	0.036*	
C28	−0.0899 (8)	0.5172 (4)	0.6444 (2)	0.0274 (13)	
H28	−0.1099	0.5527	0.6152	0.033*	
C29	0.0536 (7)	0.4668 (4)	0.6552 (2)	0.0225 (11)	
H29	0.1318	0.4680	0.6329	0.027*	
H1WA	0.208 (14)	1.035 (3)	0.668 (4)	0.10 (4)*	
H1WB	0.266 (9)	0.972 (6)	0.6345 (17)	0.05 (2)*	
H2WA	0.889 (10)	0.330 (5)	0.871 (5)	0.04 (4)*	0.50
H2WB	0.904 (10)	0.242 (4)	0.882 (4)	0.02 (3)*	0.50

### Atomic displacement parameters (Å^2^)

**Table:**

	*U*^11^	*U*^22^	*U*^33^	*U*^12^	*U*^13^	*U*^23^
Cu1	0.0096 (3)	0.0114 (3)	0.0112 (3)	−0.0016 (2)	0.0012 (2)	0.0018 (2)
Cu2	0.0172 (3)	0.0144 (3)	0.0109 (3)	0.0015 (2)	0.0023 (2)	−0.0014 (2)
Cl1	0.0143 (5)	0.0144 (5)	0.0144 (5)	−0.0022 (4)	0.0044 (4)	−0.0035 (4)
Cl2	0.0212 (6)	0.0173 (6)	0.0238 (6)	−0.0021 (5)	−0.0028 (5)	0.0082 (5)
Cl3	0.0299 (7)	0.0301 (7)	0.0190 (6)	0.0026 (6)	0.0094 (5)	0.0040 (5)
Cl4	0.0265 (6)	0.0175 (6)	0.0198 (6)	−0.0050 (5)	0.0003 (5)	−0.0031 (5)
O1	0.085 (4)	0.037 (3)	0.060 (4)	−0.001 (3)	0.052 (3)	−0.002 (3)
O2	0.023 (4)	0.025 (4)	0.028 (4)	−0.002 (3)	0.006 (3)	0.005 (3)
N1	0.0084 (17)	0.0120 (19)	0.0145 (19)	0.0006 (14)	0.0053 (14)	−0.0030 (15)
N2	0.0122 (18)	0.0108 (19)	0.0111 (18)	−0.0029 (15)	0.0020 (14)	−0.0013 (14)
N3	0.0131 (18)	0.0085 (18)	0.0154 (19)	−0.0025 (14)	0.0052 (15)	−0.0003 (15)
N4	0.016 (2)	0.014 (2)	0.018 (2)	−0.0005 (16)	0.0032 (16)	−0.0031 (16)
N5	0.0125 (19)	0.013 (2)	0.0141 (19)	0.0033 (15)	0.0028 (15)	0.0014 (15)
N6	0.019 (2)	0.018 (2)	0.013 (2)	−0.0027 (17)	−0.0012 (16)	−0.0039 (16)
C1	0.014 (2)	0.009 (2)	0.012 (2)	−0.0028 (17)	0.0033 (17)	−0.0009 (16)
C2	0.015 (2)	0.014 (2)	0.014 (2)	−0.0013 (18)	0.0048 (18)	−0.0079 (18)
C3	0.016 (2)	0.011 (2)	0.023 (3)	−0.0052 (18)	0.0107 (19)	−0.0044 (19)
C4	0.014 (2)	0.016 (2)	0.029 (3)	−0.0032 (19)	0.009 (2)	−0.015 (2)
C5	0.008 (2)	0.025 (3)	0.021 (2)	0.0015 (19)	0.0016 (18)	−0.011 (2)
C6	0.016 (2)	0.015 (2)	0.016 (2)	0.0001 (18)	0.0032 (18)	−0.0042 (18)
C7	0.012 (2)	0.014 (2)	0.013 (2)	0.0003 (17)	0.0014 (17)	−0.0003 (17)
C8	0.017 (2)	0.009 (2)	0.015 (2)	0.0028 (17)	0.0069 (18)	−0.0032 (17)
C9	0.013 (2)	0.016 (2)	0.013 (2)	0.0010 (18)	0.0013 (17)	−0.0034 (18)
C10	0.015 (2)	0.014 (2)	0.022 (3)	0.0006 (18)	0.0024 (19)	−0.0023 (19)
C11	0.017 (2)	0.014 (2)	0.031 (3)	−0.0026 (19)	0.011 (2)	0.004 (2)
C12	0.020 (2)	0.014 (2)	0.019 (2)	0.0021 (19)	0.0026 (19)	0.0045 (19)
C13	0.013 (2)	0.013 (2)	0.013 (2)	−0.0025 (17)	0.0019 (17)	−0.0034 (17)
C14	0.018 (2)	0.014 (2)	0.008 (2)	−0.0015 (18)	0.0043 (17)	−0.0011 (17)
C15	0.019 (2)	0.016 (2)	0.018 (2)	−0.0020 (19)	0.0085 (19)	−0.0039 (19)
C16	0.023 (3)	0.014 (2)	0.013 (2)	−0.0006 (19)	0.0070 (19)	−0.0024 (18)
C17	0.017 (2)	0.015 (2)	0.011 (2)	−0.0003 (18)	0.0002 (18)	−0.0008 (18)
C18	0.024 (2)	0.011 (2)	0.014 (2)	0.0023 (19)	0.0052 (19)	0.0036 (18)
C19	0.022 (2)	0.010 (2)	0.015 (2)	0.0001 (18)	0.0014 (19)	−0.0010 (18)
C20	0.030 (3)	0.019 (3)	0.017 (2)	0.003 (2)	0.000 (2)	0.001 (2)
C21	0.026 (3)	0.025 (3)	0.027 (3)	0.008 (2)	−0.004 (2)	0.001 (2)
C22	0.018 (2)	0.019 (3)	0.028 (3)	0.001 (2)	0.003 (2)	−0.009 (2)
C23	0.024 (3)	0.021 (3)	0.018 (2)	−0.004 (2)	0.007 (2)	−0.008 (2)
C24	0.015 (2)	0.018 (2)	0.021 (3)	0.0002 (19)	0.0017 (19)	−0.002 (2)
C25	0.020 (2)	0.014 (2)	0.022 (3)	−0.001 (2)	−0.002 (2)	−0.003 (2)
C26	0.018 (3)	0.017 (3)	0.036 (3)	0.002 (2)	−0.001 (2)	−0.004 (2)
C27	0.028 (3)	0.016 (3)	0.044 (4)	0.009 (2)	−0.006 (3)	−0.005 (2)
C28	0.038 (3)	0.015 (3)	0.025 (3)	0.000 (2)	−0.007 (2)	0.000 (2)
C29	0.030 (3)	0.016 (2)	0.020 (3)	−0.005 (2)	−0.001 (2)	−0.003 (2)

### Geometric parameters (Å, °)

**Table:**

Cu1—N1	1.986 (4)	C9—C10	1.372 (7)
Cu1—N3	1.996 (4)	C9—H9	0.9500
Cu1—N2	2.076 (4)	C10—C11	1.392 (7)
Cu1—Cl2	2.2832 (13)	C10—H10	0.9500
Cu1—Cl1	2.5261 (13)	C11—C12	1.382 (7)
Cu2—N4	2.002 (4)	C11—H11	0.9500
Cu2—N6	2.005 (4)	C12—H12	0.9500
Cu2—N5	2.058 (4)	C13—C14	1.517 (6)
Cu2—Cl3	2.2484 (14)	C13—H13A	0.9900
Cu2—Cl4	2.5361 (14)	C13—H13B	0.9900
O1—H1WA	0.84 (2)	C14—C15	1.524 (6)
O1—H1WB	0.84 (2)	C14—H14A	0.9900
O2—H2WA	0.84 (2)	C14—H14B	0.9900
O2—H2WB	0.83 (2)	C15—C16	1.525 (7)
N1—C6	1.339 (6)	C15—H15A	0.9900
N1—C2	1.346 (6)	C15—H15B	0.9900
N2—C1	1.473 (6)	C16—C17	1.529 (7)
N2—C7	1.486 (6)	C16—H16A	0.9900
N2—C13	1.500 (6)	C16—H16B	0.9900
N3—C8	1.342 (6)	C17—H17A	0.9900
N3—C12	1.348 (6)	C17—H17B	0.9900
N4—C23	1.337 (7)	C18—C19	1.494 (7)
N4—C19	1.354 (6)	C18—H18A	0.9900
N5—C24	1.485 (6)	C18—H18B	0.9900
N5—C17	1.487 (6)	C19—C20	1.388 (7)
N5—C18	1.488 (6)	C20—C21	1.381 (8)
N6—C29	1.343 (7)	C20—H20	0.9500
N6—C25	1.344 (7)	C21—C22	1.376 (8)
C1—C2	1.507 (6)	C21—H21	0.9500
C1—H1A	0.9900	C22—C23	1.389 (8)
C1—H1B	0.9900	C22—H22	0.9500
C2—C3	1.399 (7)	C23—H23	0.9500
C3—C4	1.386 (7)	C24—C25	1.502 (7)
C3—H3	0.9500	C24—H24A	0.9900
C4—C5	1.386 (8)	C24—H24B	0.9900
C4—H4	0.9500	C25—C26	1.390 (7)
C5—C6	1.378 (7)	C26—C27	1.387 (9)
C5—H5	0.9500	C26—H26	0.9500
C6—H6	0.9500	C27—C28	1.377 (9)
C7—C8	1.499 (7)	C27—H27	0.9500
C7—H7A	0.9900	C28—C29	1.379 (8)
C7—H7B	0.9900	C28—H28	0.9500
C8—C9	1.400 (7)	C29—H29	0.9500
			
N1—Cu1—N3	163.88 (16)	C9—C10—H10	120.4
N1—Cu1—N2	81.22 (16)	C11—C10—H10	120.4
N3—Cu1—N2	82.68 (16)	C12—C11—C10	119.1 (5)
N1—Cu1—Cl2	95.81 (12)	C12—C11—H11	120.4
N3—Cu1—Cl2	97.84 (12)	C10—C11—H11	120.4
N2—Cu1—Cl2	150.55 (12)	N3—C12—C11	121.9 (5)
N1—Cu1—Cl1	88.61 (11)	N3—C12—H12	119.1
N3—Cu1—Cl1	94.23 (12)	C11—C12—H12	119.1
N2—Cu1—Cl1	97.55 (11)	N2—C13—C14	115.7 (4)
Cl2—Cu1—Cl1	111.72 (5)	N2—C13—H13A	108.4
N4—Cu2—N6	161.75 (17)	C14—C13—H13A	108.4
N4—Cu2—N5	81.47 (16)	N2—C13—H13B	108.4
N6—Cu2—N5	81.02 (16)	C14—C13—H13B	108.4
N4—Cu2—Cl3	97.15 (13)	H13A—C13—H13B	107.4
N6—Cu2—Cl3	96.33 (13)	C13—C14—C15	109.3 (4)
N5—Cu2—Cl3	154.07 (13)	C13—C14—H14A	109.8
N4—Cu2—Cl4	94.14 (13)	C15—C14—H14A	109.8
N6—Cu2—Cl4	92.95 (13)	C13—C14—H14B	109.8
N5—Cu2—Cl4	96.58 (12)	C15—C14—H14B	109.8
Cl3—Cu2—Cl4	109.33 (5)	H14A—C14—H14B	108.3
H1WA—O1—H1WB	117 (9)	C14—C15—C16	113.6 (4)
H2WA—O2—H2WB	102 (3)	C14—C15—H15A	108.8
C6—N1—C2	118.9 (4)	C16—C15—H15A	108.8
C6—N1—Cu1	127.6 (3)	C14—C15—H15B	108.8
C2—N1—Cu1	113.3 (3)	C16—C15—H15B	108.8
C1—N2—C7	113.8 (4)	H15A—C15—H15B	107.7
C1—N2—C13	108.5 (4)	C15—C16—C17	109.4 (4)
C7—N2—C13	110.8 (3)	C15—C16—H16A	109.8
C1—N2—Cu1	103.8 (3)	C17—C16—H16A	109.8
C7—N2—Cu1	106.0 (3)	C15—C16—H16B	109.8
C13—N2—Cu1	113.9 (3)	C17—C16—H16B	109.8
C8—N3—C12	119.0 (4)	H16A—C16—H16B	108.2
C8—N3—Cu1	114.2 (3)	N5—C17—C16	117.2 (4)
C12—N3—Cu1	126.8 (3)	N5—C17—H17A	108.0
C23—N4—C19	119.3 (4)	C16—C17—H17A	108.0
C23—N4—Cu2	126.7 (4)	N5—C17—H17B	108.0
C19—N4—Cu2	114.0 (3)	C16—C17—H17B	108.0
C24—N5—C17	112.4 (4)	H17A—C17—H17B	107.2
C24—N5—C18	113.5 (4)	N5—C18—C19	108.8 (4)
C17—N5—C18	112.1 (4)	N5—C18—H18A	109.9
C24—N5—Cu2	104.3 (3)	C19—C18—H18A	109.9
C17—N5—Cu2	107.6 (3)	N5—C18—H18B	109.9
C18—N5—Cu2	106.4 (3)	C19—C18—H18B	109.9
C29—N6—C25	118.5 (5)	H18A—C18—H18B	108.3
C29—N6—Cu2	128.1 (4)	N4—C19—C20	121.0 (5)
C25—N6—Cu2	113.3 (3)	N4—C19—C18	115.7 (4)
N2—C1—C2	109.1 (4)	C20—C19—C18	123.2 (5)
N2—C1—H1A	109.9	C21—C20—C19	119.4 (5)
C2—C1—H1A	109.9	C21—C20—H20	120.3
N2—C1—H1B	109.9	C19—C20—H20	120.3
C2—C1—H1B	109.9	C22—C21—C20	119.3 (5)
H1A—C1—H1B	108.3	C22—C21—H21	120.4
N1—C2—C3	121.9 (5)	C20—C21—H21	120.4
N1—C2—C1	115.4 (4)	C21—C22—C23	119.0 (5)
C3—C2—C1	122.6 (5)	C21—C22—H22	120.5
C4—C3—C2	118.1 (5)	C23—C22—H22	120.5
C4—C3—H3	121.0	N4—C23—C22	122.0 (5)
C2—C3—H3	121.0	N4—C23—H23	119.0
C5—C4—C3	119.9 (5)	C22—C23—H23	119.0
C5—C4—H4	120.1	N5—C24—C25	109.3 (4)
C3—C4—H4	120.1	N5—C24—H24A	109.8
C6—C5—C4	118.4 (5)	C25—C24—H24A	109.8
C6—C5—H5	120.8	N5—C24—H24B	109.8
C4—C5—H5	120.8	C25—C24—H24B	109.8
N1—C6—C5	122.7 (5)	H24A—C24—H24B	108.3
N1—C6—H6	118.6	N6—C25—C26	122.5 (5)
C5—C6—H6	118.6	N6—C25—C24	115.1 (4)
N2—C7—C8	110.7 (4)	C26—C25—C24	122.4 (5)
N2—C7—H7A	109.5	C27—C26—C25	117.9 (6)
C8—C7—H7A	109.5	C27—C26—H26	121.0
N2—C7—H7B	109.5	C25—C26—H26	121.0
C8—C7—H7B	109.5	C28—C27—C26	119.9 (5)
H7A—C7—H7B	108.1	C28—C27—H27	120.0
N3—C8—C9	121.8 (4)	C26—C27—H27	120.0
N3—C8—C7	116.3 (4)	C27—C28—C29	118.7 (5)
C9—C8—C7	121.8 (4)	C27—C28—H28	120.7
C10—C9—C8	119.0 (5)	C29—C28—H28	120.7
C10—C9—H9	120.5	N6—C29—C28	122.5 (5)
C8—C9—H9	120.5	N6—C29—H29	118.8
C9—C10—C11	119.2 (5)	C28—C29—H29	118.8
			
N3—Cu1—N1—C6	−160.7 (5)	N1—C2—C3—C4	0.6 (7)
N2—Cu1—N1—C6	−163.3 (4)	C1—C2—C3—C4	178.1 (4)
Cl2—Cu1—N1—C6	−12.9 (4)	C2—C3—C4—C5	0.0 (7)
Cl1—Cu1—N1—C6	98.8 (4)	C3—C4—C5—C6	−0.4 (7)
N3—Cu1—N1—C2	25.0 (8)	C2—N1—C6—C5	0.2 (7)
N2—Cu1—N1—C2	22.3 (3)	Cu1—N1—C6—C5	−173.8 (4)
Cl2—Cu1—N1—C2	172.8 (3)	C4—C5—C6—N1	0.3 (7)
Cl1—Cu1—N1—C2	−75.5 (3)	C1—N2—C7—C8	−147.0 (4)
N1—Cu1—N2—C1	−35.2 (3)	C13—N2—C7—C8	90.5 (4)
N3—Cu1—N2—C1	145.6 (3)	Cu1—N2—C7—C8	−33.5 (4)
Cl2—Cu1—N2—C1	−121.5 (3)	C12—N3—C8—C9	−1.1 (7)
Cl1—Cu1—N2—C1	52.2 (3)	Cu1—N3—C8—C9	178.0 (3)
N1—Cu1—N2—C7	−155.4 (3)	C12—N3—C8—C7	177.0 (4)
N3—Cu1—N2—C7	25.4 (3)	Cu1—N3—C8—C7	−3.9 (5)
Cl2—Cu1—N2—C7	118.4 (3)	N2—C7—C8—N3	26.3 (6)
Cl1—Cu1—N2—C7	−68.0 (3)	N2—C7—C8—C9	−155.6 (4)
N1—Cu1—N2—C13	82.6 (3)	N3—C8—C9—C10	0.8 (7)
N3—Cu1—N2—C13	−96.7 (3)	C7—C8—C9—C10	−177.2 (4)
Cl2—Cu1—N2—C13	−3.7 (4)	C8—C9—C10—C11	0.5 (7)
Cl1—Cu1—N2—C13	170.0 (3)	C9—C10—C11—C12	−1.4 (8)
N1—Cu1—N3—C8	−15.3 (8)	C8—N3—C12—C11	0.2 (7)
N2—Cu1—N3—C8	−12.7 (3)	Cu1—N3—C12—C11	−178.8 (4)
Cl2—Cu1—N3—C8	−162.9 (3)	C10—C11—C12—N3	1.1 (8)
Cl1—Cu1—N3—C8	84.4 (3)	C1—N2—C13—C14	168.1 (4)
N1—Cu1—N3—C12	163.7 (5)	C7—N2—C13—C14	−66.4 (5)
N2—Cu1—N3—C12	166.4 (4)	Cu1—N2—C13—C14	53.0 (5)
Cl2—Cu1—N3—C12	16.1 (4)	N2—C13—C14—C15	174.9 (4)
Cl1—Cu1—N3—C12	−96.6 (4)	C13—C14—C15—C16	−171.3 (4)
N6—Cu2—N4—C23	−149.6 (5)	C14—C15—C16—C17	169.4 (4)
N5—Cu2—N4—C23	−166.1 (4)	C24—N5—C17—C16	60.6 (5)
Cl3—Cu2—N4—C23	−12.3 (4)	C18—N5—C17—C16	−68.6 (5)
Cl4—Cu2—N4—C23	97.8 (4)	Cu2—N5—C17—C16	174.8 (3)
N6—Cu2—N4—C19	32.0 (7)	C15—C16—C17—N5	−168.8 (4)
N5—Cu2—N4—C19	15.5 (3)	C24—N5—C18—C19	153.0 (4)
Cl3—Cu2—N4—C19	169.4 (3)	C17—N5—C18—C19	−78.4 (5)
Cl4—Cu2—N4—C19	−80.6 (3)	Cu2—N5—C18—C19	39.0 (4)
N4—Cu2—N5—C24	−150.3 (3)	C23—N4—C19—C20	2.2 (7)
N6—Cu2—N5—C24	34.9 (3)	Cu2—N4—C19—C20	−179.3 (4)
Cl3—Cu2—N5—C24	120.9 (3)	C23—N4—C19—C18	−175.0 (4)
Cl4—Cu2—N5—C24	−57.0 (3)	Cu2—N4—C19—C18	3.5 (5)
N4—Cu2—N5—C17	90.3 (3)	N5—C18—C19—N4	−29.2 (6)
N6—Cu2—N5—C17	−84.6 (3)	N5—C18—C19—C20	153.7 (5)
Cl3—Cu2—N5—C17	1.5 (5)	N4—C19—C20—C21	−2.9 (8)
Cl4—Cu2—N5—C17	−176.5 (3)	C18—C19—C20—C21	174.0 (5)
N4—Cu2—N5—C18	−30.1 (3)	C19—C20—C21—C22	0.9 (9)
N6—Cu2—N5—C18	155.1 (3)	C20—C21—C22—C23	1.7 (8)
Cl3—Cu2—N5—C18	−118.9 (3)	C19—N4—C23—C22	0.5 (8)
Cl4—Cu2—N5—C18	63.2 (3)	Cu2—N4—C23—C22	−177.8 (4)
N4—Cu2—N6—C29	144.3 (5)	C21—C22—C23—N4	−2.5 (8)
N5—Cu2—N6—C29	160.9 (5)	C17—N5—C24—C25	74.5 (5)
Cl3—Cu2—N6—C29	6.9 (4)	C18—N5—C24—C25	−157.0 (4)
Cl4—Cu2—N6—C29	−102.9 (4)	Cu2—N5—C24—C25	−41.7 (4)
N4—Cu2—N6—C25	−39.2 (7)	C29—N6—C25—C26	3.1 (7)
N5—Cu2—N6—C25	−22.6 (3)	Cu2—N6—C25—C26	−173.8 (4)
Cl3—Cu2—N6—C25	−176.6 (3)	C29—N6—C25—C24	−179.3 (4)
Cl4—Cu2—N6—C25	73.6 (3)	Cu2—N6—C25—C24	3.8 (5)
C7—N2—C1—C2	156.6 (4)	N5—C24—C25—N6	26.7 (6)
C13—N2—C1—C2	−79.7 (4)	N5—C24—C25—C26	−155.7 (5)
Cu1—N2—C1—C2	41.8 (4)	N6—C25—C26—C27	−1.0 (8)
C6—N1—C2—C3	−0.7 (7)	C24—C25—C26—C27	−178.4 (5)
Cu1—N1—C2—C3	174.2 (4)	C25—C26—C27—C28	−1.9 (8)
C6—N1—C2—C1	−178.3 (4)	C26—C27—C28—C29	2.6 (8)
Cu1—N1—C2—C1	−3.5 (5)	C25—N6—C29—C28	−2.3 (8)
N2—C1—C2—N1	−27.4 (5)	Cu2—N6—C29—C28	174.0 (4)
N2—C1—C2—C3	154.9 (4)	C27—C28—C29—N6	−0.5 (8)

### Hydrogen-bond geometry (Å, °)

**Table:**

*D*—H···*A*	*D*—H	H···*A*	*D*···*A*	*D*—H···*A*
O2—H2WB···Cl1^i^	0.83 (2)	2.54 (7)	3.270 (8)	147 (11)
O2—H2WA···O1^ii^	0.84 (2)	2.46 (4)	3.249 (11)	157 (10)
O1—H1WB···Cl2^iii^	0.84 (2)	2.54 (4)	3.335 (6)	159 (8)
O1—H1WA···Cl4^iv^	0.84 (2)	2.47 (3)	3.306 (6)	169 (10)

Symmetry codes: (i) −*x*+1, −*y*+1, −*z*+2; (ii) −*x*+1, *y*−1/2, −*z*+3/2; (iii) *x*, −*y*+3/2, *z*−1/2; (iv) *x*, *y*+1, *z*.
